# EPHB4 kinase–inactivating mutations cause autosomal dominant lymphatic-related hydrops fetalis

**DOI:** 10.1172/JCI85794

**Published:** 2016-07-11

**Authors:** Silvia Martin-Almedina, Ines Martinez-Corral, Rita Holdhus, Andres Vicente, Elisavet Fotiou, Shin Lin, Kjell Petersen, Michael A. Simpson, Alexander Hoischen, Christian Gilissen, Heather Jeffery, Giles Atton, Christina Karapouliou, Glen Brice, Kristiana Gordon, John W. Wiseman, Marianne Wedin, Stanley G. Rockson, Steve Jeffery, Peter S. Mortimer, Michael P. Snyder, Siren Berland, Sahar Mansour, Taija Makinen, Pia Ostergaard

**Affiliations:** 1 Lymphovascular Research Unit, Cardiovascular and Cell Sciences Institute, St. George’s University of London, London, United Kingdom (UK).; 2 Department of Immunology, Genetics and Pathology, Uppsala University, Uppsala, Sweden.; 3 Genomics Core Facility, Department of Clinical Science, University of Bergen, Bergen, Norway.; 4 Lymphatic Development Laboratory, Cancer Research UK London Research Institute, London, UK.; 5 Division of Cardiovascular Medicine and; 6 Department of Genetics, Stanford University, Stanford, California, USA.; 7 Computational Biology Unit, Department of Informatics, University of Bergen, Bergen, Norway.; 8 Division of Genetics and Molecular Medicine, King’s College London School of Medicine, Guy’s Hospital, London, UK.; 9 Department of Human Genetics, Radboud University Medical Center and Donders Centre for Neuroscience, Radboud University Medical Center, Nijmegen, Netherlands.; 10 South West Thames Regional Genetics Unit, St. George’s University of London, London, UK.; 11 Department of Dermatology, St. George’s University Hospital NHS Foundation Trust, London, UK.; 12 Discovery Sciences, RAD-Transgenics, AstraZeneca R&D, Mölndal, Sweden.; 13 Center for Medical Genetics and Molecular Medicine, Haukeland University Hospital, Bergen, Norway.

## Abstract

Hydrops fetalis describes fluid accumulation in at least 2 fetal compartments, including abdominal cavities, pleura, and pericardium, or in body tissue. The majority of hydrops fetalis cases are nonimmune conditions that present with generalized edema of the fetus, and approximately 15% of these nonimmune cases result from a lymphatic abnormality. Here, we have identified an autosomal dominant, inherited form of lymphatic-related (nonimmune) hydrops fetalis (LRHF). Independent exome sequencing projects on 2 families with a history of in utero and neonatal deaths associated with nonimmune hydrops fetalis uncovered 2 heterozygous missense variants in the gene encoding Eph receptor B4 (*EPHB4*). Biochemical analysis determined that the mutant EPHB4 proteins are devoid of tyrosine kinase activity, indicating that loss of EPHB4 signaling contributes to LRHF pathogenesis. Further, inactivation of *Ephb4* in lymphatic endothelial cells of developing mouse embryos led to defective lymphovenous valve formation and consequent subcutaneous edema. Together, these findings identify EPHB4 as a critical regulator of early lymphatic vascular development and demonstrate that mutations in the gene can cause an autosomal dominant form of LRHF that is associated with a high mortality rate.

## Introduction

Hydrops fetalis is defined as excessive fluid accumulation or edema in at least 2 fetal compartments. Nonimmune hydrops fetalis is the cause in more than 85% of cases, of which 15% have been reported to have a lymphatic-related abnormality (1). In 20% of nonimmune hydrops fetalis cases, the cause is not known. Lymphatic-related (nonimmune) hydrops fetalis (LRHF) has been included in a subgroup of primary lymphedemas under the umbrella term generalized lymphatic dysplasia (GLD) by Connell et al. (2). In this classification, GLD was defined as lymphedema associated with systemic or visceral involvement (including hydrops fetalis), even if the lymphedema was not widespread. The GLD group includes patients with a widespread developmental abnormality of the lymphatic system, often presenting prenatally with hydrothoraces or nonimmune hydrops fetalis.

Hennekam syndrome (OMIM 235510) is an example of a GLD that is inherited in an autosomal recessive manner. Mutations in collagen and calcium binding EGF domains 1 (*CCBE1*) and FAT atypical cadherin 4 (*FAT4*) have been identified as causal (3–5). Another recessively inherited form of GLD with a high incidence of LRHF has recently been reported as caused by mutations in piezo-type mechanosensitive ion channel component 1 (*PIEZO1*) (6), adding to the genetic heterogeneity of the GLD group.

We have recently ascertained 2 families with a history of nonimmune hydrops and postnatal lymphatic dysfunction, but with a pattern suggestive of autosomal dominant inheritance and with sporadic occurrence in one of the families. We have identified heterozygous inactivating mutations in the kinase domain of Eph receptor B4 (*EPHB4*) as causative for this condition. This suggests not only that LRHF/GLD is genetically heterogeneous, but also that it should be considered in both dominant and recessive forms. The importance of EPHB4 for lymphatic vascular development is further supported by analysis of a genetic mouse model with lymphatic endothelial-specific deletion of *Ephb4*.

## Results

### Genetic analysis of LRHF identifies causative mutations in EPHB4.

**Figure 1:**
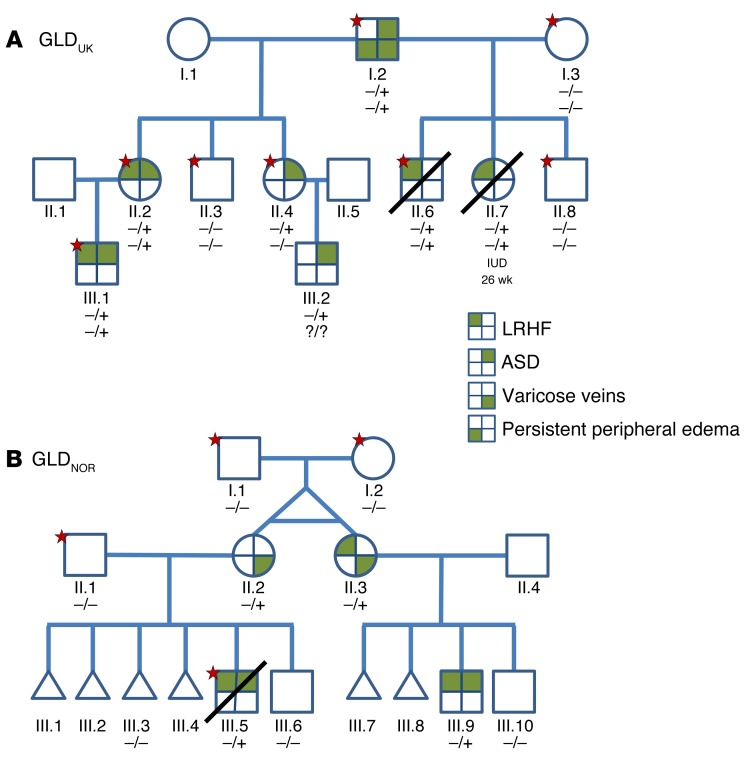
Mutations in *EPHB4* cause LRHF. (**A**) Pedigree of GLD_UK_ family. (**B**) Pedigree of GLD_NOR_ family. Stars indicate which samples have been exome sequenced. Genotypes indicated by minus signs (–) represent the WT allele, and plus signs represent (+) the mutant allele. Top row of genotypes in GLD_UK_ genogram shows *EPHB4*, and bottom row of genotypes shows *MIER2*. Triangles, first trimester miscarriages; IUD, intrauterine death. GLD_NOR_:III.3 had trisomy 18.

**Figure 2:**
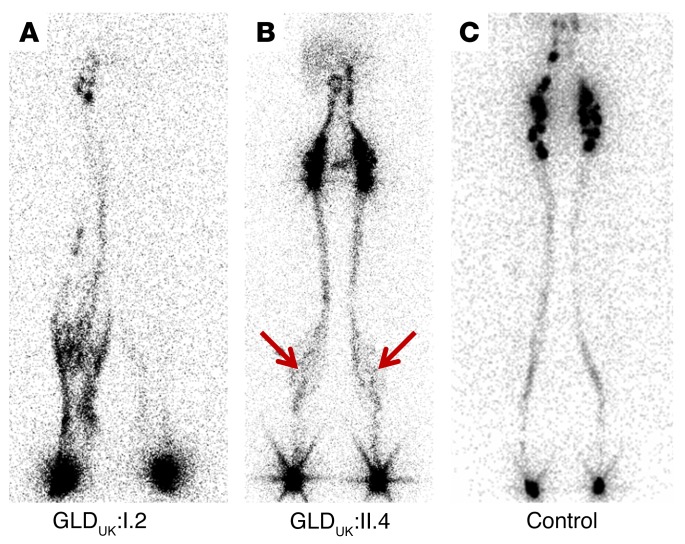
Imaging of the lymphatic system in LRHF. **Anterior view of lower limb lymphoscintigraphy 2 hours after injection with radionuclide.** (**A**) GLD_UK_:I.2, rerouting through skin and superficial tissues in the right leg and markedly reduced transport in the left leg. (**B**) GLD_UK_:II.4, normal uptake of tracer in the lymph nodes in the groin area, but with some rerouting in the calves (seen as the dark shading; arrows). (**C**) Unaffected subject with symmetrical transport of radionuclide within collecting lymph vessels in the leg.

We report 2 multigenerational families (one from Norway [GLD_NOR_] and one from the UK [GLD_UK_]) (Figure 1). Clinical findings in these families included antenatal nonimmune hydrops fetalis or bilateral hydrothoraces and neonatal chylothoraces of variable severity (Table 1) as well as a high number of first trimester miscarriages (GLD_NOR_). The nonimmune hydrops fetalis is associated with a high mortality, but babies surviving the neonatal period improve with time, with spontaneous resolution of the hydrops and pleural effusions. Only 1 (GLD_UK_:I.2) of 5 adults had developed peripheral edema of the lower extremities in adolescence (Figure 2), but 3 of the 5 adults (GLD_NOR_:II.2, GLD_NOR_:II.3 and GLD_UK_:I.2) had varicose veins. Although GLD_UK_:II.2 and GLD_NOR_:II.2 had no clinical evidence of peripheral lymphedema at the time of this writing, lymphoscintigraphy showed mild bilateral impairment of lymph drainage with retention of tracer in the lower limbs in both. In addition, GLD_UK_:II.2 had an atrial septal defect (ASD), diaphragmatic hernia, and cystic hygroma at birth. There appeared to be an association with ASD in both families (*n* = 7).

Sanger sequencing identified no pathogenic variants in the genes known to be associated with congenital primary lymphedema (i.e., *CCBE1*, *VEGFR3*, and *VEGFC*) in the UK proband (GLD_UK_:I.2). Whole-exome sequencing (WES) was performed on the family to identify pathogenic variants. When filtering the WES data of the UK family for variants in genes known to have relevance to the lymphatic system and lymphangiogenesis, an unreported variant (NM_004444.4: c.2216G>A, p.Arg739Glu) in *EPHB4* was identified. Initially, it was thought that the variant did not fully cosegregate with the disorder status in the family (Figure 1A), as 2 clinically unaffected family members were found to carry the variant (GLD_UK_:II.4 and GLD_UK_:III.2). Neither of these presented with hydrops fetalis, but both were later diagnosed with an ASD. The next step of the analysis was to apply a specific autosomal dominant inheritance filter criterion, and *MIER2* was the only gene that fulfilled that (Figure 1A). However, the *MIER2* variant (NM_017550.1: c.865C>T, p.Arg289Trp) has been reported as a SNP (rs148482834), with a heterozygous genotype observed at a frequency of 0.001.

Meanwhile, an independent study of a GLD_NOR_ family was undertaken. In this family, the condition initially appeared to be sporadic in monozygotic twins (GLD_NOR_:II.2 and (GLD_NOR_:II.3), who both had subcutaneous edema at birth that resolved in infancy (Table 1). GLD_NOR_:II.3 required ventilation and thoracentesis for bilateral chylothoraces. Both sisters had sons with nonimmune hydrops. One died at 1.5 days of age; the other was moribund in the neonatal period, but the edema eventually resolved. Both sons also had an ASD. Three genes (protein tyrosine phosphatase, non-receptor type 11 [*PTPN11*], forkhead box C2 [*FOXC2*], and *VEGFR3*) were ruled out in GLD_NOR_:II.2 by Sanger sequencing, and a high-resolution microarray comparative genomic hybridization (CGH) (Affymetrix 6.0) was normal in GLD_NOR_:II.3. When analyzing the WES data, the only gene to fulfil an autosomal dominant model with de novo occurrence was *EPHB4*. Sanger sequencing of additional family members showed all affected family members carried the variant (c.2345T>G, p.Ile782Ser) (Figure 1B). GLD_NOR_:II.2 and GLD_NOR_:II.3 were both found to be mosaic for this variant, and GLD_NOR_:II.2 had a lower mutation load than her twin sister, GLD_NOR_:II.3, consistent with her milder neonatal presentation (Supplemental Figure 1; supplemental material available online with this article; doi:10.1172/JCI85794DS1).

As *MIER2* had shown perfect cosegregation in GLD_UK_, the WES data of GLD_NOR_:III.5 was scrutinized, but no variants were found in *MIER2*, and the coverage was found to be of sufficient depth and quality. For good measure, GLD_NOR_:III.5 was also screened by Sanger sequencing for all exons of *MIER2*; still, no variant was identified. Neither *EPHB4* variants had been previously reported in public databases or in 900 in-house controls, and it was therefore concluded that mutations in *EPHB4* were the likely cause of the LRHF/GLD phenotype seen in these 2 families despite the variable expression observed.

### LRHF-associated mutations lead to inactive EPHB4 kinase.

**Figure 3:**
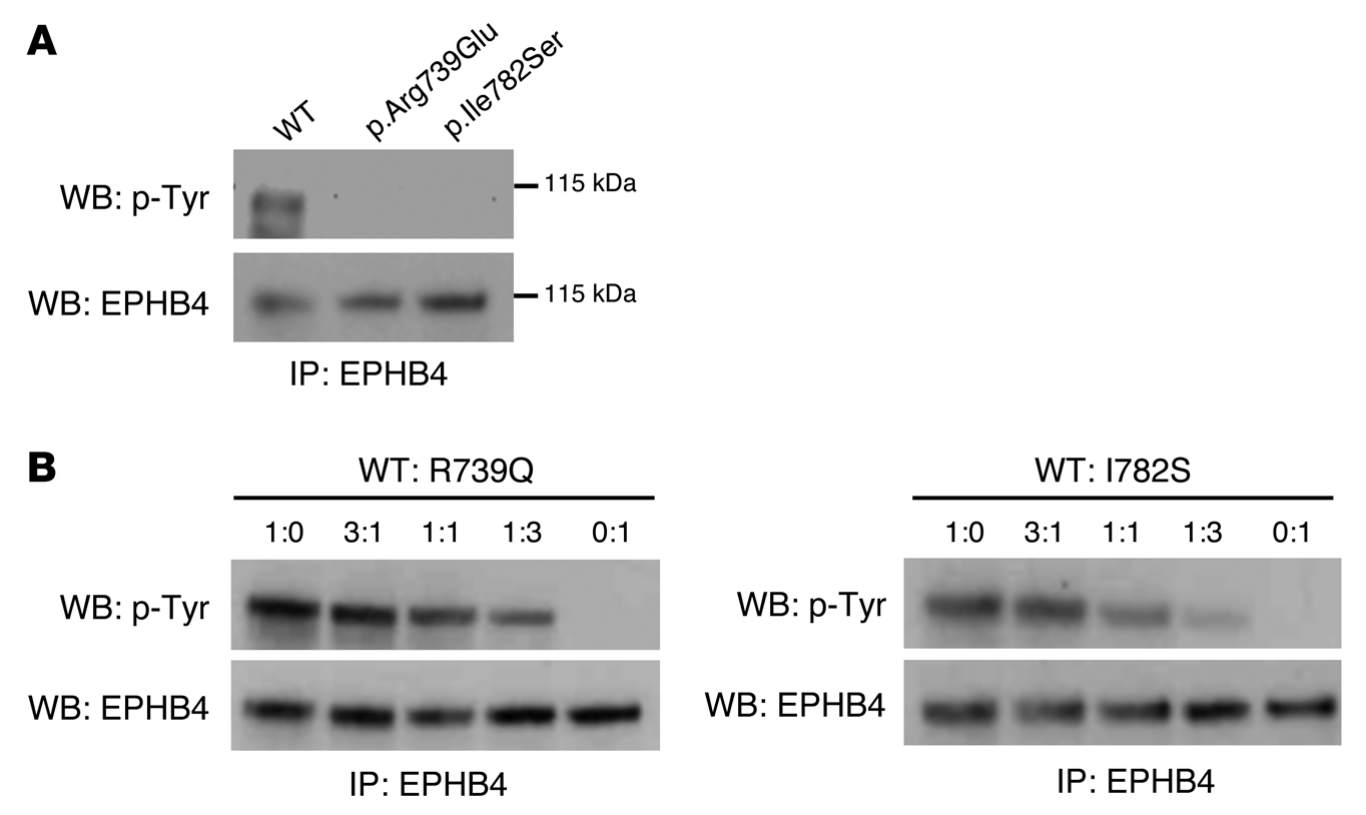
Effect of p.Arg739Glu and p.Ile782Ser mutations on EPHB4 tyrosine phosphorylation in HEK293T cells. (**A**) HEK293T cells were transfected with expression plasmids for EPHB4 WT and p.Arg739Glu and p.Ile782Ser mutants. Receptor phosphorylation was analyzed by immunoprecipitation and Western blotting using anti–p-tyrosine (p-Tyr, upper panel) and EPHB4 (lower panel) antibodies. The positions of molecular mass markers (in kDa) are indicated to the right of the gels. (**B**) EPHB4 WT and p.Arg739Glu and p.Ile782Ser mutants were cotransfected into HEK293T cells in different ratios of WT to mutant plasmid. Receptor phosphorylation was analyzed as above. One representative experiment (*n* = 3) is shown.

**Figure 4:**
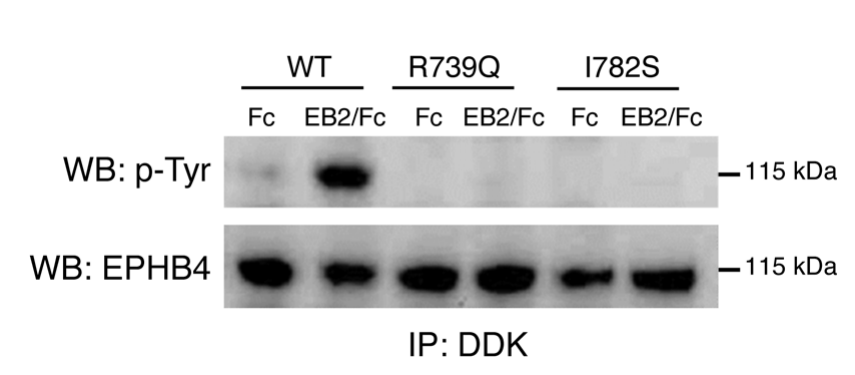
Effect of p.Arg739Glu and p.Ile782Ser mutations on EPHB4 tyrosine phosphorylation in LECs after Ephrin B2 stimulation. LECs were transfected with expression plasmids for Myc-DDK–tagged EPHB4 WT and p.Arg739Glu and p.Ile782Ser mutants. The cells were stimulated with 1 μg/ml clustered Ephrin B2/Fc (EB2/Fc) or Fc alone. Receptor phosphorylation was analyzed by immunoprecipitation with anti-DDK antibody and Western blotting using anti–p-tyrosine (upper panel) and EPHB4 (lower panel) antibodies. The positions of molecular mass markers (in kDa) are indicated to the right of the gels. One representative experiment (*n* = 2) is shown.

EPHB4 binds the transmembrane Ephrin B2. Binding of Ephrin B2 to EPHB4 stimulates phosphorylation and activates downstream signaling cascades (7, 8). The 2 *EPHB4* mutations (p.Arg739Glu and p.Ile782Ser) occur at highly conserved residues located in the tyrosine kinase domain of the EPHB4 protein (Supplemental Figure 2 and Supplemental Figure 3A). Moreover, p.Arg739Glu is located within the catalytic loop HRD (His-Arg-Asp) motif, also highly conserved in many tyrosine kinases (Supplemental Figure 3B). To investigate the effect of the mutations identified in the patients with LRHF, corresponding expression constructs for WT and mutant proteins by site-directed mutagenesis were generated and analyzed for their phosphorylation activity after transient transfection in HEK293T cells. The tyrosine phosphorylation levels of WT, p.Arg739Glu, and p.Ile782Ser mutants were analyzed by immunoprecipitation and Western blotting with anti-EPHB4 and anti–p-tyrosine–specific antibodies. Mutant proteins showed no phosphorylation (Figure 3A), suggesting that both mutations alter EPHB4 signaling in patients with LRHF/GLD. To test the possibility that the mutations were interfering with the phosphorylation state of the WT protein, different ratios of WT and mutant proteins were cotransfected and phosphorylation of the receptor analyzed as described above. Results showed no dominant negative effect, as phosphorylation of the total receptor decreased when increasing amounts of mutated receptor were cotransfected (Figure 3B). Furthermore, Ephrin B2–dependent EPHB4 activation was evaluated in lymphatic endothelial cells (LECs) after expression of Myc-DDK–tagged EPHB4. To distinguish phosphorylation levels of Myc-DDK–tagged exogenous expressed WT and mutant EPHB4 from endogenous expressed WT EPHB4, an anti-DDK antibody was used for the immunoprecipitation and isolation of only the overexpressed forms of Myc-DDK–tagged EPHB4. Ephrin B2 treatment increased phosphorylation levels of WT Myc-DDK–tagged EPHB4, but not mutant proteins (Figure 4), confirming the negative effect of both mutations on the receptor activity after ligand stimulation in LECs.

### EPHB4 deficiency in mice results in subcutaneous edema and abnormal lymphatic development.

**Figure 5:**
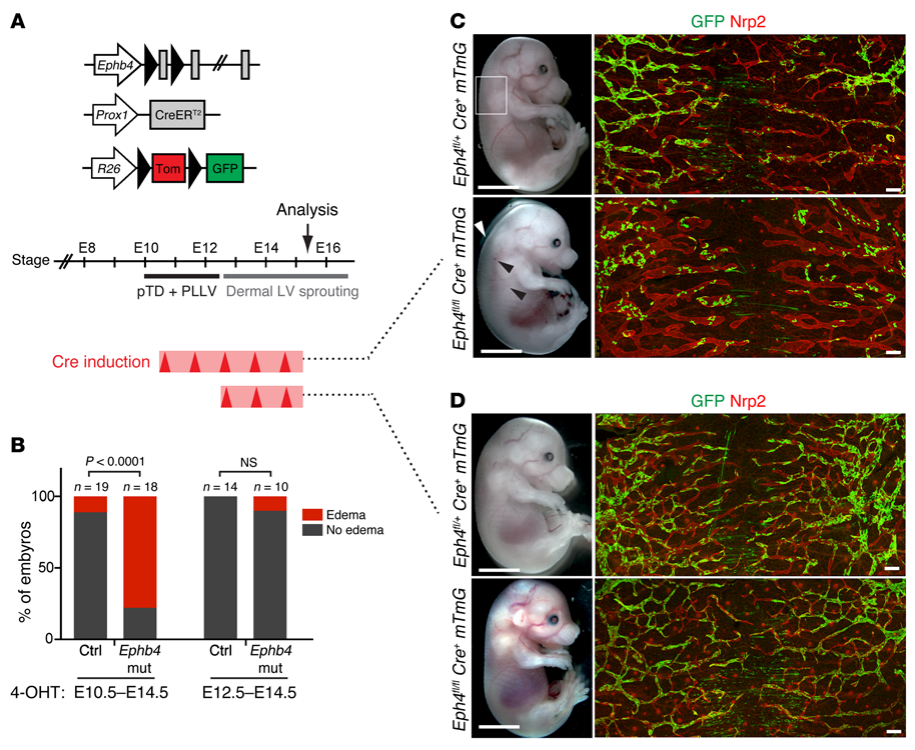
Early embryonic deletion of *Ephb4* leads to subcutaneous edema and abnormal dermal lymphatic vasculature. (**A**) Schematic of the transgenes and 4-OHT administration (*Cre* induction; red arrowheads) schedule used for *Ephb4* deletion in the lymphatic vasculature. Timing of primitive lymphatic vessel formation and time point for analysis are indicated. PLLV, peripheral longitudinal lymphatic vessel; LV, lymphatic vessel. (**B**) Edema in E15.5 control and *Ephb4* mutant embryos after different 4-OHT treatments. *P* values determined by Fisher’s exact test. (**C** and **D**) Left panels: E15.5 *Ephb4^fl/+^* and *Ephb4^fl/fl^ Prox1-CreER^T2^ R26-mTmG* embryos. Most mutants treated with 4-OHT at E10.5–E14.5 showed subcutaneous edema (white arrowhead) and blood-filled lymphatic vessels (black arrowheads). Boxed area indicates the area of the skin imaged on the right. Right panels: whole-mount immunofluorescence of E15.5 thoracic skin for NRP2 (red) and GFP (green) to stain lymphatic vessels and gene-targeted cells, respectively. Scale bars: 200 μm (**C** and **D**).

Ephrin B2/EPHB4 signaling is critically required for the development of the cardiovascular system during early embryogenesis (9, 10). Ephrin B2 and EPHB4 are also essential for lymphatic vessel remodeling and valve formation during late embryonic and early postnatal development (11, 12), but whether they have an earlier role in lymphatic vessel morphogenesis that could explain *Ephb4* loss of function–induced LRHF/GLD in humans is not known. Whole-mount immunofluorescence analysis confirmed the previously reported venous and lymphatic endothelial-specific expression of EPHB4 in embryonic skin and mesenteries (Supplemental Figure 4, A and B). To assess the potential contribution of lymphatic endothelial EPHB4 loss of function to LRHF/GLD, we deleted *Ephb4* specifically in the lymphatic vasculature using tamoxifen-inducible *Prox1-CreER^T2^* mice crossed with a conditional *Ephb4^fl^* line (Figure 5A and Supplemental Figure 5). The mice were further crossed with the *R26-mTmG* double reporter to monitor *Cre* activity and to label gene-deleted cells with GFP (*Ephb4^fl/fl^ Prox1-CreER^T2^ R26-mTmG* mice, referred to here as *Ephb4* mutants). *Ephb4* deletion was induced from the earliest stage of lymphatic development by administration of 4-hydroxytamoxifen (4-OHT) for 5 consecutive days starting at E10.5 (Figure 5A). At E15.5, a high proportion of mutant embryos showed subcutaneous edema (Figure 5, B and C). In addition, a proportion of dermal lymphatic vessels contained blood in 71% of edematous mutant embryos (*n* = 14), but not in nonedematous mutants (*n* = 5) or in control embryos (*n* = 20) (Figure 5C and data not shown). Whole-mount immunofluorescence of the skin revealed tortuous and dilated dermal lymphatic vessels in the *Ephb4* mutants (Figure 5C). Notably, abnormal vessel morphology was also observed in vessels that showed a low contribution of GFP^+^ (i.e., *Ephb4*-deficient cells) (Figure 5C), suggesting that edema and/or blood filling of lymphatic vessels secondarily caused vessel dilation. In support of a non–cell-autonomous effect of early embryonic deletion of *Ephb4* on dermal lymphatic vasculature, inactivation of *Ephb4* from E12.5, when dermal lymphatic vessel formation begins (ref. 13 and Figure 5A), resulted in normal vasculature despite efficient gene targeting (Figure 5D). These results suggest E10–E12 as a critical time-window for EPHB4 function during lymphatic development.

### Ephb4 is required for the formation of lymphovenous and lymphatic valves.

**Figure 6:**
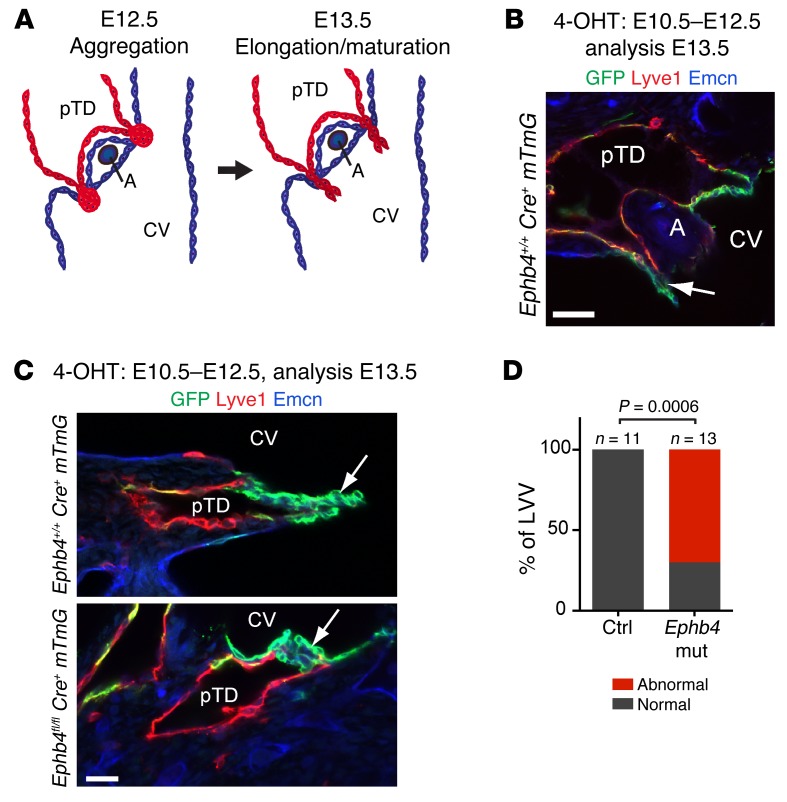
Early embryonic deletion of *Ephb4* leads to a failure of LVV formation. (**A**) Schematic representation of LVV formation. CV, cardinal vein; A, artery. Adapted with permission from *Developmental Biology* (ref. 16; Creative Commons user license available at http://www.sciencedirect.com/science/article/pii/S0012160615301032). (**B**) Whole-mount immunofluorescence of a transverse section of E13.5 *Ephb4^+/+^ Prox1-CreER^T2^ R26-mTmG* embryo for indicated proteins. GFP shows efficient *Cre*-mediated recombination in the LVV that extends to the lumen of the cardinal vein (arrow). (**C**) Whole-mount immunofluorescence of E13.5 LVVs showing extension of the valve leaflets to the lumen of the cardinal vein (arrow) in control (upper panel, *n* = 11 out of 11) but not in *Ephb4* mutant (*Ephb4* mut) embryo (lower panel, *n* = 9 out of 13). (**D**) Quantification of LVV morphology in control and *Ephb4* mutant embryos. Normal, elongated leaflets; abnormal, no leaflets. *P* value determined by Fisher’s exact test. Scale bars: 50 μm (**B**); 25 μm (**C**).

Previous studies have shown that between E10.5 and E13.5, formation of specialized lymphovenous valves (LVVs) occurs at the connection sites between the primordial thoracic duct (pTD) and the cardinal vein (refs. 14–16 and Figure 6A). It was therefore reasoned that edema in *Ephb4* mutants might be due to defective LVVs leading to inefficient lymph drainage. To investigate this, we induced *Cre* recombination in the developing LVVs in *Ephb4^fl/fl^ Prox1-CreER^T2^ R26-mTmG* embryos by 4-OHT treatment between E10.5 and E12.5. Analysis of immunostained transverse vibratome sections of E13.5 control embryos showed preferential and efficient targeting of the dual LVVs by the *Prox1-CreER^T2^* transgene, while pTD endothelium exhibited mosaic labeling (Figure 6B). Control LVVs (11 out of 11) consisted of 2 well-defined leaflets extending to the lumen of the cardinal vein (Figure 6, C and D, and Supplemental Video 1). In contrast, the majority of EPHB4-deficient LVVs (9 out of 13) did not show extended leaflets, but instead consisted of abnormal clusters of GFP^+^ cells (Figure 6, C and D, and Supplemental Video 2).

Interestingly, studies using a function-blocking antibody and a chemical genetic approach showed that EPHB4 kinase signaling regulates lymphatic valve formation (11), while genetic studies have demonstrated an important function for its ligand, Ephrin B2, in the formation of both lymphatic and venous valves (12, 17). Using our genetic loss-of-function model, we confirmed the essential role of EPHB4 in lymphatic valve morphogenesis. Deletion of *Ephb4* during embryonic valve formation led to a complete absence of valves that form in control mesenteries by E18.5 by LECs expressing high levels of PROX1 (Supplemental Figure 4C). In addition, early postnatal deletion of *Ephb4* led to a complete loss of lymphatic valves (Supplemental Figure 4D). These results demonstrate a critical role of *Ephb4* in the formation and early postnatal maintenance of lymphatic valves and highlight conserved mechanisms regulating the formation of valves at different anatomical sites.

## Discussion

This study identifies the EPHB4 receptor tyrosine kinase as a critical regulator of early lymphatic vessel development and a causative gene for LRHF and primary lymphedema. We have shown here that kinase-inactivating mutations in *EPHB4* can produce a lymphatic phenotype in humans that can be classified as LRHF/GLD. However, this phenotype shows highly variable expression. Some individuals present with severe in utero swelling, which may cause perinatal demise (or fully resolve to become completely asymptomatic), others with no edema but only an ASD. This phenotype can be distinguished from the majority of Hennekam syndrome cases, in which the swelling presents in the antenatal period but persists throughout life (3–5). The large number of miscarriages in GLD_NOR_ may well be related to this disorder. In this regard, it is of interest that EPHB4 and Ephrin B2 have been shown to be instrumental in human placental development (18). Invasive cytotrophoblasts use the EPHB4 expression on veins to ensure that migration of these cells into EPHB4-expressing uterine veins is limited and instead biased toward the arterial side of the circulation (19). Expression of EPHB4 at half the levels normally encountered may disturb the complex migration patterns seen in the process of placentation. A failure of the invasive cytotrophoblasts to take on an arterial phenotype is suspected as leading to the loss of pregnancy during the late first or early second trimester (19). Perinatal deaths were also of a higher frequency in the autosomal recessive form of LRHF/GLD caused by *PIEZO1* mutations but, in this condition, were probably related to the hydrops fetalis (6).

Two GLD_UK_ family members (GLD_UK_:II.4 and her son, GLD_UK_:III.2) carry the variant, but have no clinical history of pre- or postnatal swelling. On lymphoscintigraphy (GLD_UK_:II.4), quantification showed entirely normal levels of transport of lymph within the legs, but imaging was suggestive of rerouting through skin and superficial tissues rather than a main lymphatic tract as seen in the control (Figure 2B). She had a small ASD and, interestingly, her son had large, multiple ASDs requiring surgical closure. Variable expression has been observed in other primary lymphedemas, e.g., PIEZO1-related LRHF/GLD (6).

Like other forms of GLD (4, 6), this condition presents antenatally with nonimmune hydrops or pleural effusions. The swelling may completely resolve with no residual lymphatic phenotype, which is similar to observations in the recently identified *PIEZO1*-related GLD (6). However, the report of 1 affected individual with bilateral lower limb edema (GLD_UK_:I.2) with abnormal lymph scans suggests that there may be residual, lymphatic weakness in the survivors. Further studies will be needed to investigate the specific nature and extent of the lymphatic dysfunction in these patients.

LEC-specific deletion of *Ephb4* in mouse embryos led to subcutaneous edema and abnormal lymphatic vessel morphology, thus recapitulating aspects of the human LRHF phenotype. Temporal analysis of EPHB4 function demonstrated a critical requirement of EPHB4 during early stages of lymphatic development. Specifically, we found that EPHB4 regulates the formation of LVVs that are critical for efficient lymphatic function by maintaining unidirectional flow of lymph into blood (14, 20, 21). We additionally confirmed the previously reported critical role of EPHB4 in both formation and maintenance of lymphatic valves (11). Lymphatic valve defects are, however, an unlikely cause of in utero swelling due to their late embryonic development (22, 23). We postulate, therefore, that defective LVV formation, caused by the lack of EPHB4, could contribute to the LRHF seen in the GLD_NOR_ and GLD_UK_ patients. In agreement with this, defective LVVs were recently demonstrated in mouse models of primary lymphedemas caused by loss of function of FOXC2, connexin 37, and GATA2 (16, 24).

Edema in the mouse embryos lacking EPHB4 specifically in the lymphatic endothelia appears to be milder than that observed in the patients, suggesting that defective LVVs may only partially explain human LRHF/GLD. It is well known that EPHB4 is also expressed in venous and capillary endothelium (9, 10), and therefore, the impact of the mutations on the venous system needs to be considered. In accordance with this hypothesis, some of the patients (GLD_NOR_:II.2 and GLD_NOR_:II.3; GLD_UK_:I.2) showed varicose veins, which would be consistent with a valve defect in the venous system and would support the contribution of vascular deficiency to the observed phenotype. Together with the varicose veins, the ASD observed in the patients could also be an important part of this phenotype. Both have been reported in lymphedema distichiasis syndrome with a frequency of approximately 7% (ASDs) (25) and 100% (varicose veins) (26).

Kinase activity is critical for EPHB4 forward signaling in lymphatic endothelia (11). Our in vitro data show that *EPHB4* mutants carrying the LRHF-associated mutations p.Arg739Glu and p.Ile782Ser are kinase dead, but do not have a dominant negative effect on WT protein. In contrast, *VEGFR3* mutants in Milroy disease have a slower turnover (27), which may affect the signaling capacity of the WT tyrosine kinase receptor due to accumulation of mutant receptors on the cell surface. Unlike typical receptor tyrosine kinases that dimerize upon ligand stimulation, Eph receptors form higher order clusters, with cluster size being an important determinant of the quality and strength of cellular response (28, 29). Inclusion of a kinase-dead receptor may thus significantly weaken the signaling strength of higher order clusters and thereby alter cellular responses. EPHB2 receptor–mediated endocytosis requires the kinase activity of the receptor (30), so kinase-dead EPHB4 could also show defective endocytosis, influencing clustering dynamics and cellular responses. Further studies will aim to investigate those and other functional consequences of the LRHF/GLD-associated mutations.

In conclusion, we report on kinase-inactivating *EPHB4* mutations in 11 individuals from 2 extended family pedigrees presenting with a phenotypic spectrum from severe, lethal nonimmune hydrops to ASD only. The inheritance pattern is typical of autosomal dominant inheritance with variable expression. Using a genetic mouse model, we have further shown that *Ephb4* deficiency in lymphatic endothelium leads to defective LVVs, which may critically contribute to edema formation in LRHF/GLD patients. This is the first report, to our knowledge, of a human phenotype associated with *EPHB4* mutations and also, we believe, the first report of an autosomal dominant form of LRHF.

## Methods

### Exome sequencing.

For GLD_UK_, sequencing libraries were made following the protocol from Roche/Nimblegen’s SeqCap EZ Exome Library v2.0 kit. The libraries were then sequenced on HiSeq2000 (Illumina) machines. Sequence reads were aligned to the reference genome (hg19) using Novoalign (Novocraft Technologies). Duplicate reads, resulting from PCR clonality or optical duplicates, and reads mapping to multiple locations were excluded from downstream analysis. Depth and breadth of sequence coverage were calculated with custom scripts and the BedTools package (31).

All variants were annotated using a custom annotation pipeline. Single-nucleotide substitutions and small indel variants were identified and quality filtered within the SamTools software package (32) and in-house software tools (33). Variants were annotated with respect to genes and transcripts with the Annovar tool (34). Variants were filtered for novelty by comparing them with dbSNP135 and 1000 Genomes SNP calls and with variants identified in 900 control exomes (primarily of European origin), which were sequenced and analyzed by the same method. Summary statistics for the exome sequencing are given in Supplemental Tables 1 and 2. Analysis of the exome-variant profiles was performed under a model of a rare autosomal dominant disorder; this model required one previously unobserved heterozygous variant for all affected individuals.

For GLD_NOR_, the sequencing analysis was performed using the SOLID 5500xl platform (Life Technologies). Exon sequences were enriched by SureSelect Human All Exon v5 (Agilent Technologies), which targets approximately 21,500 human genes, covering a total of 50 Mb of genomic sequence. Read alignment and variant calling were performed with Lifescope v1.3 software. All variants were annotated using a custom annotation pipeline. Variants from the exome were filtered for known variants in dbSNP, intronic and UTR variants, synonymous variants, and variants in our in-house database. Variants with fewer than 5 variation reads were also omitted. Summary statistics for the exome sequencing are given in Supplemental Table 3.

### Confirmation sequencing.

Samples of available family members were analyzed by Sanger sequencing. Primers were designed for the coding regions and associated splice sites for exons 13 and 14 of *EPHB4* and all exons of *MIER2* using Primer3 software (35) or ExonPrimer (https://www.helmholtz-muenchen.de/). Primer sequences are listed in Supplemental Table 4. PCR products were sequenced using BigDye Terminator v1.1 and v3.1 chemistry (Life Technologies) and an ABI3130xla Genetic Analyzer or 3730 DNA analyzer (Life Technologies). Sequencing traces were visually inspected in Finch TV v1.4 (Geospiza Inc.) and SeqScape Software v2.5 (Life Technologies).

The twins in GLD_NOR_ were also examined for mosaicism by Sanger sequencing on DNA extracted directly from blood (new sample), urine, saliva, and skin biopsy (fibroblasts).

The variants from the 2 families have been submitted to the Leiden Open Variation Database (LOVD^3^) (genomic variants 0000095140 [c.2216G>A; p.(Arg739Gln)] and 0000095141 [c.2345T>G; p.(Ile782Ser)]; http://databases.lovd.nl/shared/genes/EPHB4).

### Site-directed mutagenesis of EPHB4 constructs.

The human EPHB4 mammalian expression plasmids pCMV6-XL6-EPHB4 (SC117357) and Myc-DDK–tagged pCMV6-Entry-EPHB4 (RC208559) were obtained from OriGene and used as templates for site-directed mutagenesis using the QuikChange II XL Site-Directed Mutagenesis Kit (Agilent). All primers were designed using QuikChange Primer Design (Agilent) and are listed in Supplemental Table 5. All constructs were verified by DNA sequencing.

### Cell culture and transfection.

HEK293T cells (provided by Tris McKay, St. George’s University of London) were maintained in DMEM supplemented with 10% FBS. Transfection of HEK293T cells was performed with GeneJuice Transfection Reagent (Merk) following the manufacturer’s protocol; 6 × 10^5^ cells/well were seeded in 6-well plates the day before transfection, and then they were transfected with 3 μl GeneJuice and 1 μg of DNA. Human dermal LECs (C-12217) were obtained from PromoCell and maintained in supplemented endothelial cell growth medium MV2 (C-22022, PromoCell) containing recombinant human VEGF-C (2179-VC-025, R&D Systems). Transfection of LECs was performed with Viromer YELLOW (VY-01LB-01, Lipocalyx) following the manufacturer’s recommendations. 2 × 10^5^ cells/well were seeded in fibronectin-coated (F1141, Sigma-Aldrich) 6-well plates the day before transfection and then transfected with 1 μg of DNA. For both cell types, lysates were collected 24 hours after transfection and subjected to immunoprecipitation and Western blot analysis.

### Ligand activation of EPHB4 receptor.

Ephrin B2/Fc (7397-EB-050, R&D Systems) or Fc fragment alone (CSB-NP005401h, Stratech) was clustered by incubation with a goat anti-human IgG Fc antibody (40C-CR2022G-FIT, Stratech) at a 1:2 ratio for 1 hour at 4°C. Eighteen hours after transfection, LECs were serum starved for 6 hours and then treated with 1 μg/ml clustered Ephrin B2/Fc or Fc alone for 30 minutes before cell lysate collection.

### Immunoprecipitation and Western blot analysis.

For immunoprecipitation of overexpressed WT and mutant EPHB4, transfected cells were harvested in 100 μl of lysis buffer (20 mM Tris pH 7.5, 150 mM NaCl, 0.5%Triton X-100) supplemented with protease and phosphatase inhibitors (Sigma-Aldrich). After clarification by centrifugation, 100 μg of total HEK293T cell protein lysate was incubated with 0.8 μg of goat anti-human EPHB4 antibody (AF3038, R&D Systems) overnight at 4°C. After incubation with protein A sepharose beads (Sigma-Aldrich) for 4 hours at 4°C, immune complexes were precipitated by centrifugation. 100 μl of total LEC protein lysate was incubated with 4 μg of mouse anti-DDK antibody (clone OTI4C5, TA50011, Origene) overnight at 4°C and immune complexes precipitated with protein G sepharose beads (Sigma-Aldrich). Immunoprecipitates were separated by SDS-PAGE and transferred to PVDF membranes. Immunoblot analysis was performed with goat anti-human EPHB4 (AF3038, R&D Systems) and mouse anti-phosphotyrosine (clone 4G10, 05-321, Millipore) antibodies. All uncropped Western blots are shown in Supplemental Figures 6 and 7.

### Mouse lines.

We generated *Ephb4^fl/fl^ Prox1-CreER^T2^ R26-mTmG* mice as follows: *R26-mTmG* mice were acquired from the Jackson Laboratory (36). *Prox1-CreER^T2^* mice were previously described (17). For the generation of the *Ephb4^fl^* line, a conditional KO strategy, flanking *Ephb4* exons 2 and 3 with *LoxP* sites, was used to target the *Ephb4* locus. The targeting vector was built using homologous recombination in bacteria (37). A C57BL/6 mouse BAC served as a template for the extraction of the homology arms of the targeting vector. The targeting vector contained a *frt* flanked neomycin phosphotransferase (Neo) selectable marker cassette. After linearization, the targeting construct was electroporated into AZX1, a C57BL/6JOlaHsd-derived embryonic stem cell line. PCR screens and Southern blot analyses revealed clones that had undergone the desired homologous recombination event. Several of these clones were expanded and injected into BALB/cOlaHsd blastocysts to generate chimeric males that were then bred to C57BL/6JOlaHsd females. Black-coated offspring were genotyped on both sides of the homology arms for correct integration into the *EphB4* locus. The Neo selectable marker cassette, which was flanked by *frt* sites, was deleted after subsequent breeding to mice expressing *flp* recombinase under the CAG promoter.

For embryonic induction of *Cre* recombination, 4-OHT (Sigma-Aldrich) was dissolved in peanut oil (10 mg/ml). 1 mg was administered to pregnant females by intraperitoneal injection at the indicated developmental stages. For induction during early postnatal development, mice were administered at P1 and P2 with 50 μg of 4-OHT dissolved in ethanol by intraperitoneal injection. All strains were maintained and analyzed on a C57BL/6J background.

### Immunofluorescence.

For whole-mount immunostaining, tissues were fixed in 4% paraformaldehyde (PFA) for 2 hours at room temperature, followed by permeabilization in 0.3% Triton X-100 PBS (PBSTx) and blocking in PBSTx 3% milk. Primary antibodies were incubated at 4°C overnight in blocking buffer. After washing several times, the samples were incubated with fluorochrome-conjugated secondary antibodies in blocking buffer for 2 hours before further washing and mounting in Mowiol. The following primary antibodies were used: goat anti-mouse Ephb4 (AF446, R&D Systems), goat anti-human VE-cadherin (sc-6458, Santa Cruz Biotechnology Inc.), rabbit anti-human Prox1 (generated against human PROX1 C terminus; 567–737 aa), rabbit anti-GFP (A11122, Invitrogen), chicken anti-GFP (Ab13970, Abcam), rat anti–PECAM-1 (553370, BD Biosciences — Pharmingen), rat anti-endomucin (sc-65495, Santa Cruz Biotechnology Inc.) and rabbit anti-Lyve1 (103-PA50AG, Reliatech). Secondary antibodies conjugated to AF488, Cy3, or AF647 were obtained from Jackson ImmunoResearch. Controls were littermate embryos and mice of the following genotypes: *Ephb4^fl/+^ Prox1-CreER^T2+^ R26-mTmG^+^, Ephb4^fl/fl^ Prox1-CreER^T2–^ R26-mTmG^+^*, or *Ephb4^fl/fl^ Prox1-CreER^T2–^ R26-mTmG^–^*.

For analysis of LVVs, 100-μm coronal vibratome sections of E13.5 embryos were cut and stained as described above. Single-plane images of the valve were taken where the valve was clearly visible. Only valves that appeared to be intact were included in the classification. Five control embryos (*Ephb4^fl/+^ Prox1-CreER^T2+^ R26-mTmG^+^* and *Ephb4^+/+^ Prox1-CreER^T2+^ R26-mTmG^+^*) were cut, and 11 valves were imaged and included in the quantification. For mutant embryos (*Ephb4^fl/fl^ Prox1-CreER^T2+^ R26-mTmG^+^*), 13 valves from 9 embryos were imaged.

### Image acquisition.

Stereomicroscope images were acquired using a Leica MZ16F fluorescence microscope with a Leica DFC420C camera and Leica Microsystems software. Confocal images were acquired using a Zeiss LSM 780 confocal microscope and Zen 2009 software or a Leica SP8 confocal microscope and Leica Application Suite X software. Images shown in Figure 5, C and D, were acquired as 4 × 2 tile scans with a HCX PL APO CS ×10/0.40 dry objective. Images in Figure 6 were acquired using a Fluotar VISIR ×25/0.95 water objective. Images in Supplemental Figure 4A were taken with a HC PL APO CS2 63×/1.30 GLYC objective; images in Supplemental Figure 4B (P6 mesentery) were taken using a C-Aphochromat ×40/1.20 W Korr M27 objective, and images in Supplemental Figure 4, B (E18 mesentery), C, and D, were acquired using a Plan-Apochromat ×20/0.8 Ph2. All confocal images represent maximum intensity projections of z-stacks except images in Figure 6.

### Lymphoscintigraphy.

Lymphoscintigraphy is the imaging of the lymphatic system by injecting radioactive isotope (technetium-99m) into the web spaces between the toes and/or fingers and quantification of uptake into the inguinal lymph nodes for foot injections and axillary nodes for hand injection after 2 hours with a gamma camera.

### Statistics.

*P* values representing differences in the proportion of edematous and nonedematous mutant versus control littermates (Figure 5B) and the proportion of normal and abnormal LVVs (Figure 6D) were calculated using χ^2^/Fisher’s exact test (2-tailed). A *P* value of less than 0.05 was considered significant.

### Study approval.

Subjects in this study were recruited through genetic and lymphovascular clinics in Norway and the UK. Ethical approval for this study was obtained from the Norwegian Regional Committees for Medical and Health Research Ethics and the West (REC ref: 2011/2453) and South West London Research Ethics Committee (REC Ref: 05/Q0803/257). Written, informed consent was obtained for all subjects. All affected individuals and family members underwent a detailed physical examination. Animal studies were approved by the UK Home Office and the Uppsala Laboratory Animal Ethical Committee.

## Author contributions

SMA, IMC, SB, SJ, TM, and PO designed the research study. SMA, IMC, EF, RH, AV, SL, HJ, and CK conducted experiments. MAS, KP, SL, CG, and AH provided the NGS pipeline. SMA, IMC, RH, AV, SL, SB, SJ, SM, TM, and PO analyzed data. GA, GB, KG, MW, JWW, SB, PSM, SGR, SM, TM, and PO provided reagents, material, and patients. SMA, IMC, SB, SJ, SM, TM, and PO wrote the manuscript. PSM, SGR, SJ, SM, MPS, TM, and PO supervised the research.

## Supplementary Material

Supplemental data

Supplemental Video 1

Supplemental Video 2

## Figures and Tables

**Table 1:**
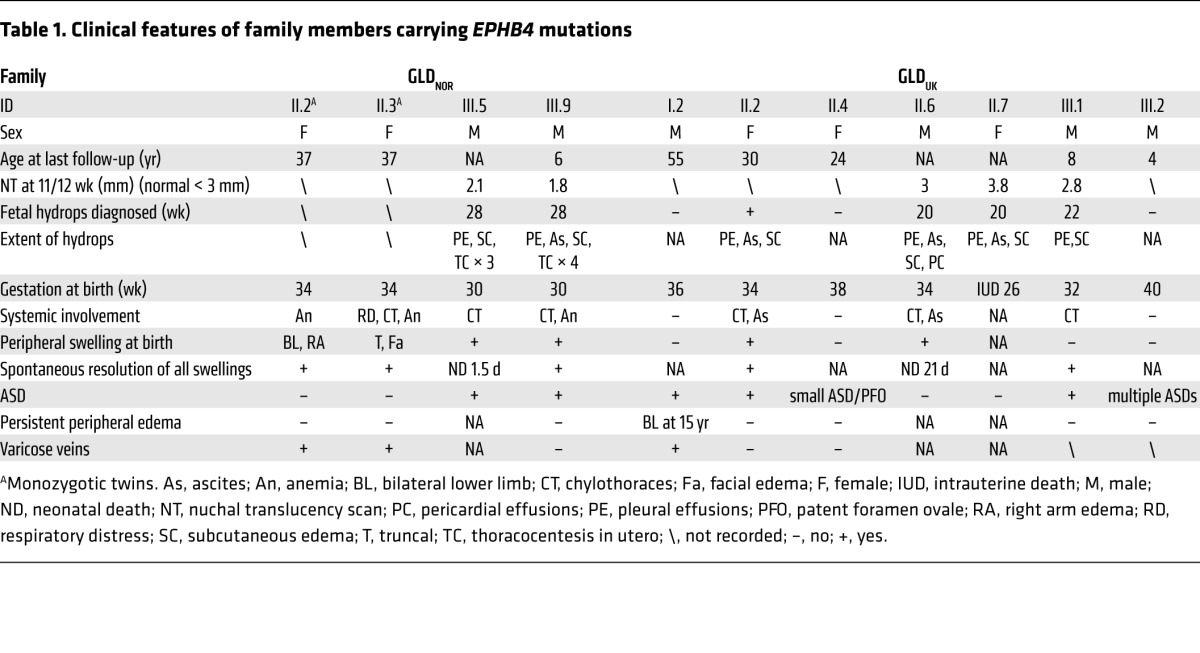
Clinical features of family members carrying *EPHB4* mutations
