# Supplementary material for: EPHB4 kinase–inactivating mutations cause autosomal dominant lymphatic-related hydrops fetalis
Source: J Clin Invest. 2016 Jul 11;126(8):3080–8. doi: 10.1172/JCI85794 (PMC4966301; doi:10.1172/JCI85794)
Supplement: Supplemental data [file JCI85794.sd.pdf]

Supplemental material

EPHB4 kinase inactivating mutations cause autosomal dominant lymphatic-related hydrops fetalis

Silvia Martin-Almedina,[1*] Ines Martinez-Corral,[2*] Rita Holdhus,[3] Andres Vicente,[4,5] Elisavet Fotiou,[1] Shin Lin,[6,7] Kjell Petersen,[8] Michael A Simpson,[9] Alexander Hoischen,[3,10] Christian Gilissen,[10] Heather Jeffery,[1] Giles Atton,[11] Christina Karapouliou,[1] Glen Brice,[11] Kristiana Gordon,[12] John W Wiseman,[13] Marianne Wedin,[13] Stanley G Rockson,[6] Steve Jeffery,[1] Peter S Mortimer,[1] Michael P Snyder,[7] Siren Berland,[14] Sahar Mansour,[11] Taija Makinen,[2] Pia Ostergaard[1]

Supplemental Figures

Supplemental Figure 1: Mosaicism in twin sisters in Norwegian family, GLD$_{NOR}$

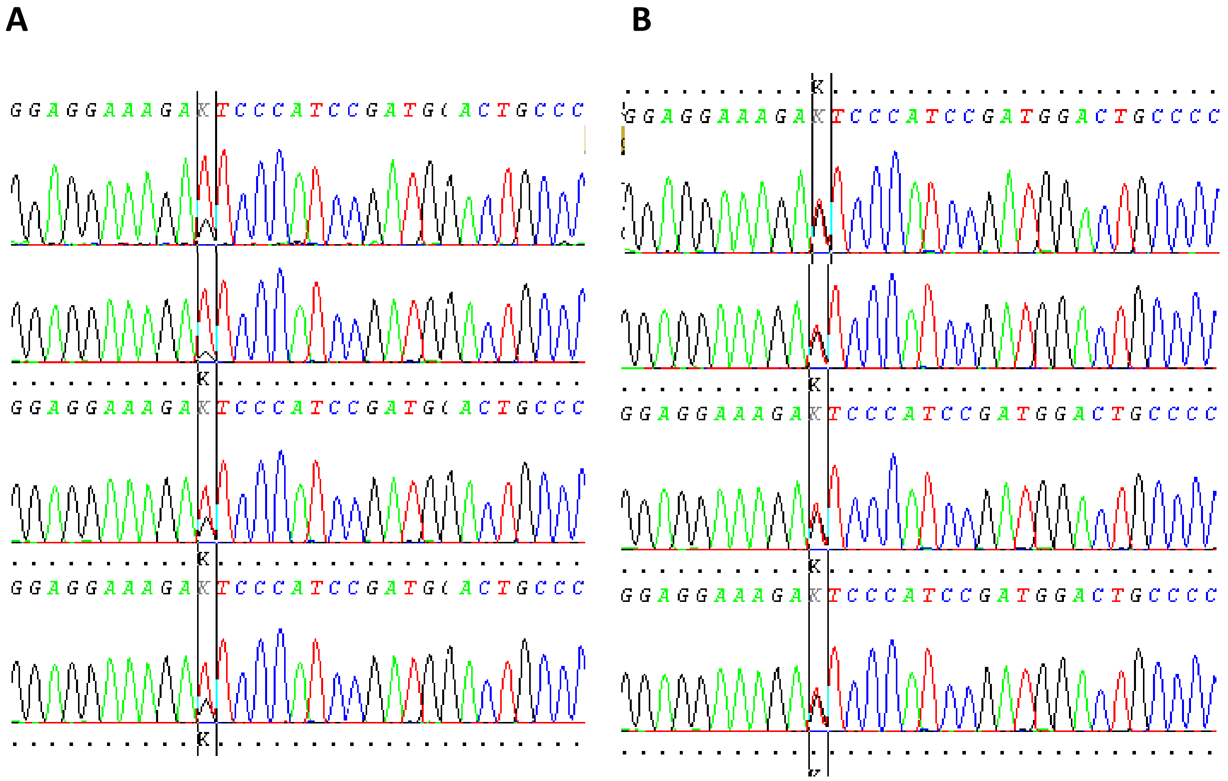

Sanger sequencing traces of c.2345T>G *EPHB4* variant from DNA extracted from fibroblasts (skin biopsy) (upper panel), blood (2nd panel), saliva (3rd panel) and urine (lower panel). **(A)** Patient GLD$_{NOR}$:II.2 has following ratio of wildtype and mutant alleles in fibroblasts: 87/13; blood: 69/31; saliva: 72/28; and urine: 74/26. **(B)** Patient GLD$_{NOR}$:II.3 showed a 50/50 ratio of wildtype and mutant allele in all 4 samples.

Supplemental Figure 2: Location of the identified mutations in EPHB4

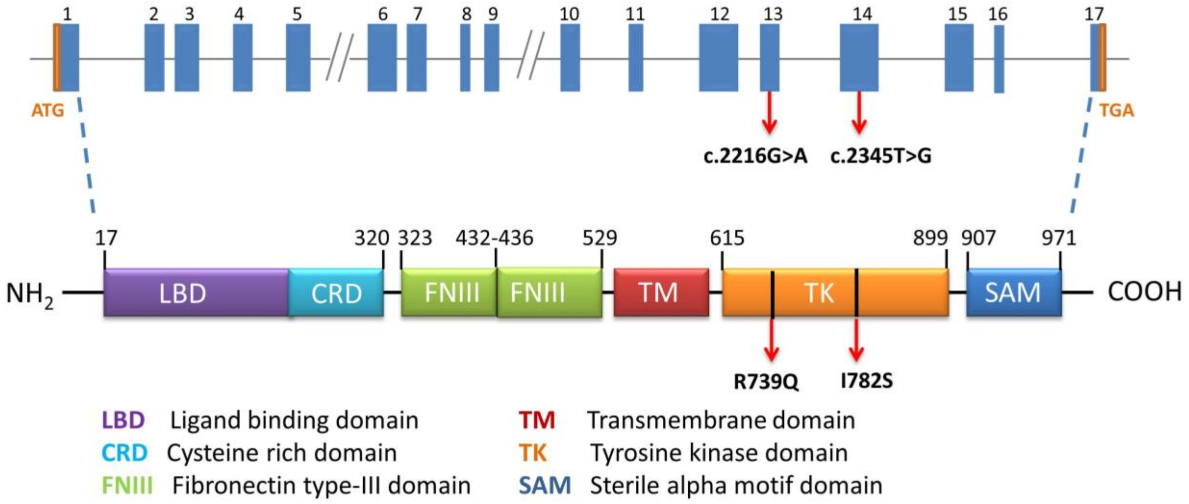

Top panel shows the genomic organisation of *EPHB4* with full blue boxes representing exons while introns are indicated by lines. The bottom panel represents the protein domains of EPHB4. The red arrows indicate the position of the identified mutations in relation to exons (upper panel) and protein level (lower panel). Both mutations are located in the tyrosine kinase domain of EPHB4.

Supplemental Figure 3: Conservation of the residues altered by the *EPHB4* mutations in the two families

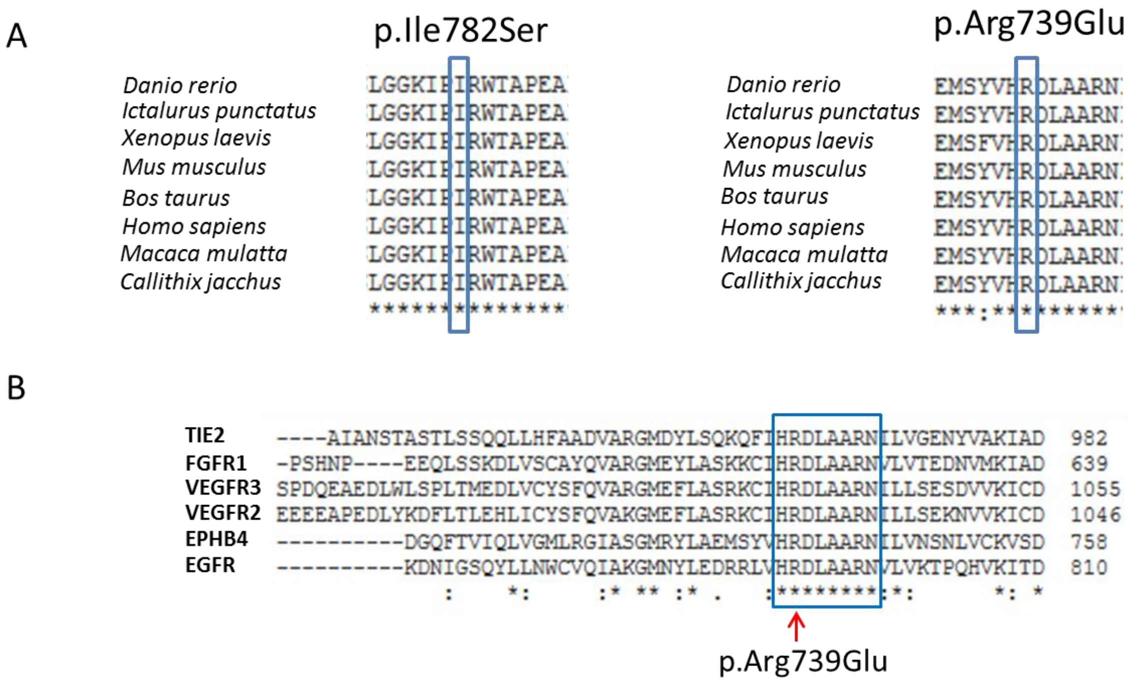

(A) The EPHB4 protein sequences of *Danio rerio* (AC# NP_571489.1), *Xenopus laevis* (NP_001129644.1), *Mus musculus* (AC# AAK28823.1), *Bos taurus* (AC# NP_001193197.1), *Macaca mulatta* (AC# AFJ71030.1), *Homo sapiens* (AC#EAL23820.1), *Ictalurus punctatus* (AC# AHH39016.1) and *Callithrix jacchus* (AC# JAB11637.1) were aligned with Clustal Omega (www.clustal.org/omega/*)*. Asterisks denote highly conserved residues between the different species. **(B)** Different human tyrosine kinase receptors protein sequences aligned with Clustal Omega. Conserved catalytic loop (HRD; His-Arg-Asp) residues are highlighted.

Supplemental Figure 4: EphB4 in lymphatic valve formation and maintenance

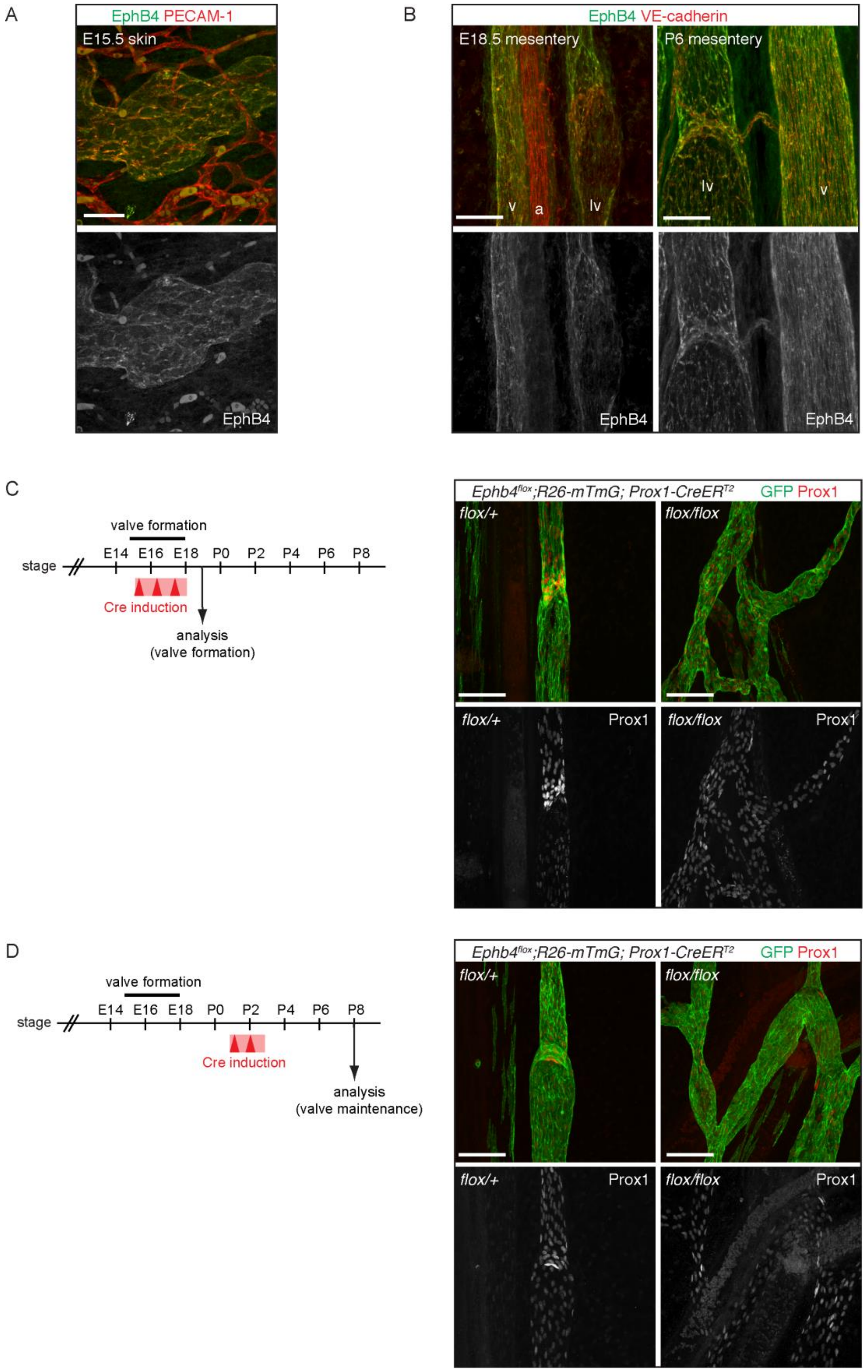

(A, B) Whole-mount immunofluorescence of embryonic skin (A) and embryonic and early postnatal mesenteries (B) for indicated antibodies. Single channel images for EphB4 are shown. v = vein, a = artery, lv = lymphatic vessel. **(C, D)** On the left: Schematic of the 4-OHT administration (Cre induction; red arrowheads) schedule used for *Ephb4* deletion. Timing of lymphatic valve formation in the mesentery and time-point for analysis are indicated. On the right: Whole-mount immunofluorescence of mesenteric vessels. Lymphatic valves are identified in control vessels (n=3 for each stage) by clusters of $Prox1^{high}$ cells (red) that are absent in the mutants (n=3 for each stage). GFP (green) shows efficient Cre-mediated recombination in lymphatic endothelium. Single channel images for Prox1 staining are shown. Scale bars: 50 µm (A and B), 100 µm (C and D).

Supplemental Figure 5: *Ephb4* gene targeting strategy

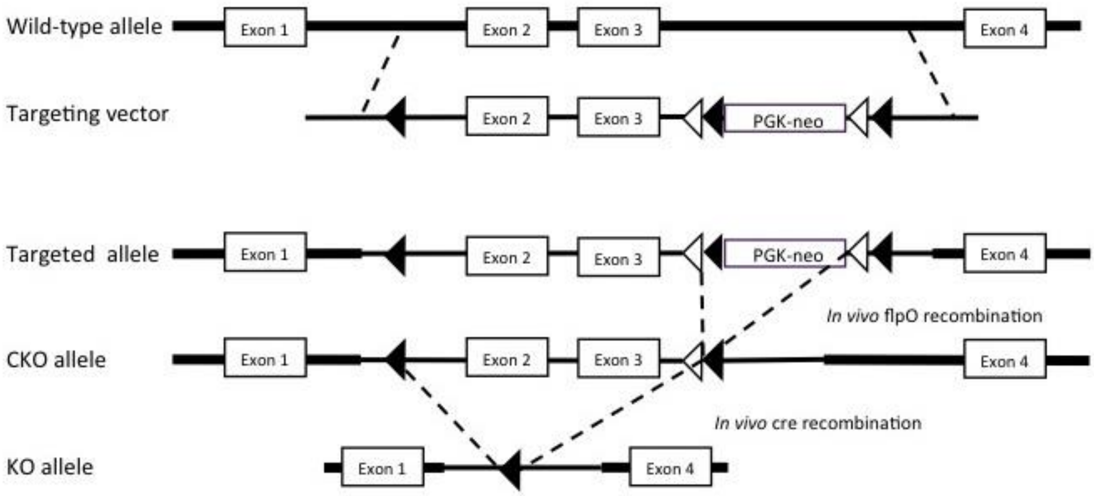

Schematic diagram of the targeting strategy used to generate *Ephb4* conditional knock-out mice. Closed triangles indicate *loxP* sites and open triangles indicate *frt* sites. KO = knock-out, CKO = conditional knock-out.

Supplemental Figure 6: Effect of p.Arg739Glu and p.Ile782Ser mutations on EPHB4 tyrosine phosphorylation in HEK293T cells

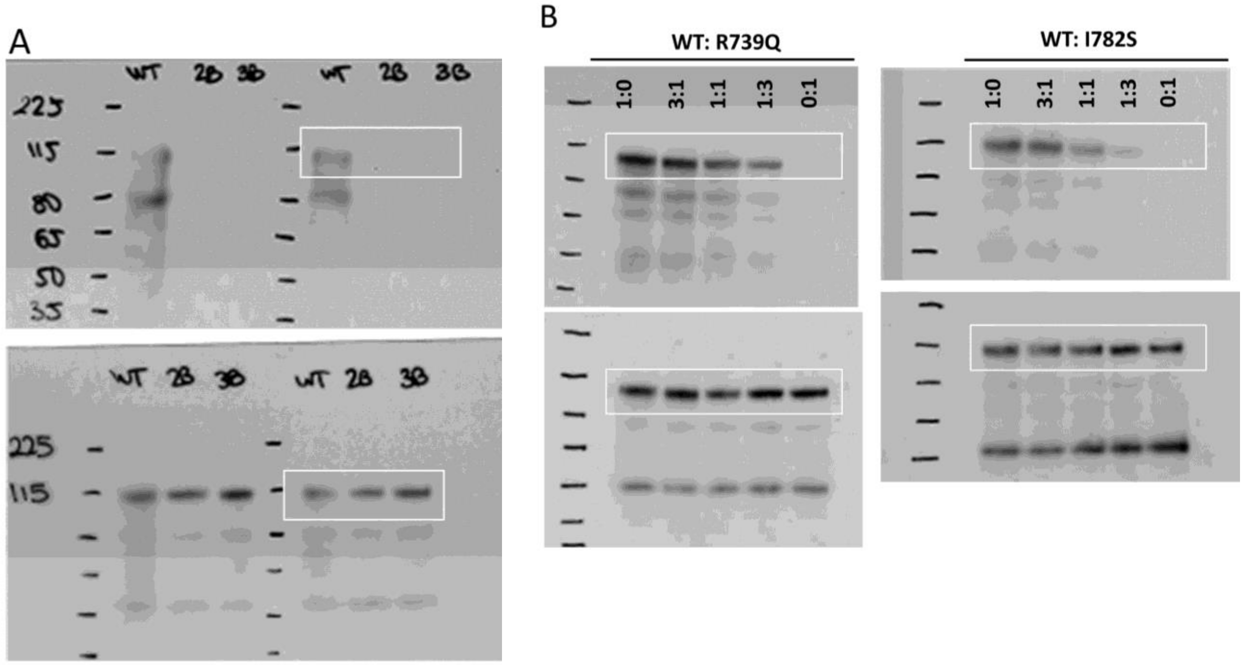

Original western blots with white outline indicating which sections have been used in Figure 3 in the paper. **(A)** The top panel is the P-Tyr and the lower panel is the EPHB4. **(B)** Western blot for the two mutants when cotransfected. The top panel is the P-Tyr and the lower is the EPHB4. See legend of Figure 3 for interpretation.

Supplemental Figure 7: Effect of p.Arg739Glu and p.Ile782Ser mutations on EPHB4 tyrosine phosphorylation in LECs after EphrinB2 stimulation

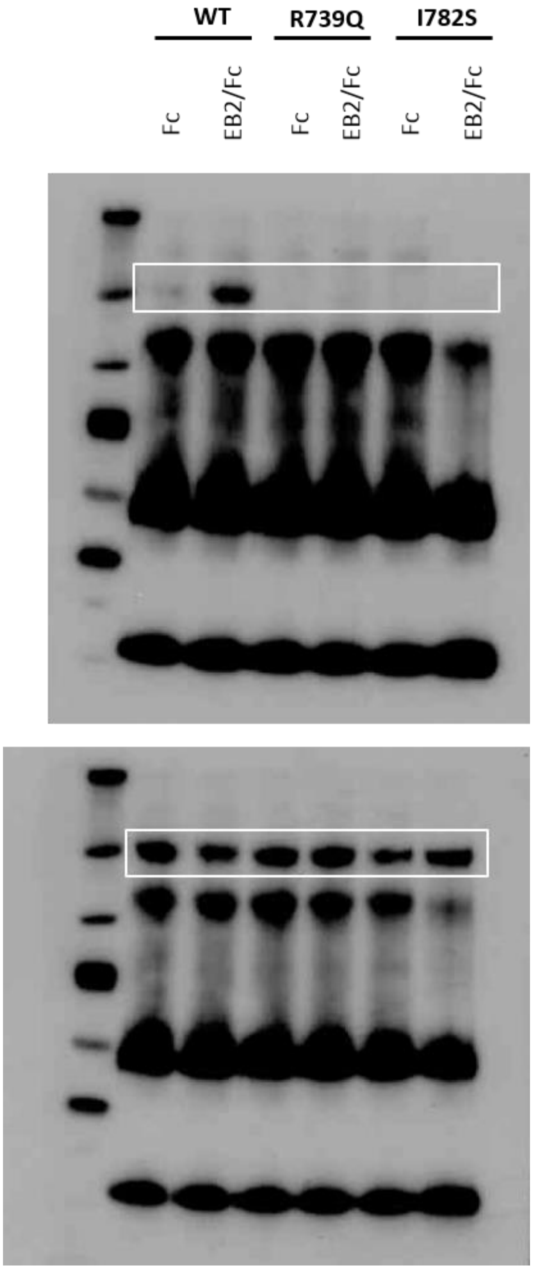

Original western blots with white outline indicating which sections have been used in Figure 4 in the paper. The top panel is the P-Tyr and the lower panel is the EPHB4. See legend to Figure 4 for interpretation.

Supplemental Tables

Supplemental Table 1: Summary statistics for exome sequencing - mapping and coverage in GLD_{UK}

Sample ID	II.2	II.3	II.8	III.1	I.2	I.3	II.4	II.6
**Total sequence reads**	97721033	106939040	104164431	76801562	109911842	114728053	158666687	140524223
**Reads mapped to target**	71043264	81598116	82290420	56506591	82600976	87073991	104029656	104817018
**% reads in target regions +/- 150bp**	80.76	83.61	86.37	82.23	81.75	86.01	72.18	88.69
**Mean coverage**	125.97	145.91	147.75	99.57	149.41	151.78	185.64	190.11
**% of GENCODE exome covered > 1x**	96.64	96.55	96.61	96.2	97.1	96.65	97.67	98.16
**% of GENCODE exome covered > 5x**	92.95	93.04	93.52	92.16	93.73	93.21	93.99	97.22
**% of GENCODE exome covered > 10x**	90.53	90.75	91.5	89.02	91.68	90.92	92.03	96.52
**% of GENCODE exome covered > 20x**	86.11	86.65	87.93	82.91	88.1	86.77	88.98	94.86

Supplemental Table 2: Summary statistics for exome sequencing – variant calling in GLD_{UK}

	II.2	II.3	II.8	III.1	I.2	I.3	II.4	II.6
All variants	23838	23566	23739	23603	24183	24155	24252	24776
Heterozygous	14876	14499	14514	14474	15322	15326	14854	14904
Homozygous	8962	9067	9225	9129	8861	8829	9398	9872
Coding variants	21019	20801	21015	20848	21368	21344	21436	21896
Heterozygous	13117	12788	12869	12812	13544	13562	13159	13209
Homozygous	7902	8013	8146	8036	7824	7782	8277	8687
Splice variants	2819	2765	2724	2755	2815	2811	2816	2880
Heterozygous	1759	1711	1645	1662	1778	1764	1695	1695
Homozygous	1060	1054	1079	1093	1037	1047	1121	1185
Nonsynonymous SNVs	9692	9493	9582	9599	9844	9909	9825	9869
Heterozygous	6159	5854	5869	5968	6293	6385	6066	5943
Homozygous	3533	3639	3713	3631	3551	3524	3759	3926
Synonymous SNVs	10528	10497	10609	10420	10687	10612	10759	11043
Heterozygous	6452	6414	6509	6347	6695	6673	6565	6696
Homozygous	4076	4083	4100	4073	3992	3939	4194	4347
Stop-loss SNVs	13	13	13	12	13	10	13	13
Heterozygous	8	9	10	11	9	6	9	10
Homozygous	5	4	3	1	4	4	4	3
Stop-gain SNVs	85	78	77	85	86	78	81	76
Heterozygous	68	61	60	69	72	60	65	60
Homozygous	17	17	17	16	14	18	16	16
Deletions	177	162	161	172	176	174	176	239
Heterozygous	120	103	88	107	122	115	118	149
Homozygous	57	59	73	65	54	59	58	90
Insertions	128	136	145	144	148	134	151	211
Heterozygous	68	69	72	68	91	70	75	110
Homozygous	60	67	73	76	57	64	76	101
Frameshift deletions	65	63	67	69	61	66	65	70
Heterozygous	38	34	33	37	34	36	38	37
Homozygous	27	29	34	32	27	30	27	33
Frameshift insertions	42	45	45	47	47	46	53	65
Heterozygous	23	18	17	21	27	16	25	29
Homozygous	19	27	28	26	20	30	28	36
TS:TV ratio	2.92	2.98	3.01	2.82	2.94	2.86	2.92	2.99
Heterozygous	2.84	2.92	3.01	2.67	2.88	2.76	2.86	3.03
Homozygous	3.06	3.08	3	3.08	3.06	3.03	3.01	2.94

Numbers of variants of different classes identified by exome sequencing in the eight exome sequenced GLD_{UK} cases. SNV, single nucleotide variant; TS:TV, transition:transversion ratio.

Supplemental Table 3: Summary statistics for exome sequencing - mapping and coverage of GLD_{NOR}

**Sample ID**	D09-2964/GLD_{NOR}:III.5	60563108/GLD_{NOR}:I.2	63114310/GLD_{NOR}:I.1	60557433/GLD_{NOR}:II.1
**Library ID**	2013_079_BC15_L	2013_080_BC16_L	2013_081_BC10_L	2013_082_BC1_L
**Average Depth of Coverage within Targets**	74.76	79.77	85.56	94.01
**Number of Variants called**	46659	47423	47155	47217
**Total Mapped reads (%)**	92.90%	92.80%	93.40%	93.20%

Supplemental Table 4: Primer sequences (5'- 3') for exons 13 and 14 of *EPHB4* (NM_004444.4) and all exons of *MIER2* (NM_017550.1)

Gene	Exon	Primer Sequence - Forward	Primer Sequence - Reverse	Product Size
MIER2	1	CAAGATCAGGGTCCAGCAGA	GGCCTCAGAGCTCCAGAG	367bp
	2	GAGACAAAGCCCCTTCCCT	GTCCACAGCTCAGCCAGG	313bp
	3	GCCTCATCCTTAGGGCTTCA	TCAGCTACTAGGACAGCATCT	387bp
	4	GGGAGTTGCTATGGGTGTGA	GCCTCCTCTCACTGAACACT	346bp
	5	GCAGAATGGGAATAGCAGCC	GAGTTCTTGGCCTGCATCAG	353bp
	6	TGTGCCCATCTGTTTACCCA	GCAGAACCACAGTCCGGA	467bp
	7	CCCGTAAGCCTGCCACAG	ACCAGACTGTCCAAGGATCTG	246bp
	8	CTGAGTTGGCTGCTTTGCTT	CCCCTTCTGACCTGAGTAGC	397bp
	9	TCGCTGGATGGTATAGACAGT	GTTCTTTCCATGCACACGGA	482bp
	10	GGTGATCCGAGGTGAGCAG	CAGAGCCTTGTTCTTGTGGG	565bp
	11	TATCTGAGAGTGGTGGGTGC	CAGACAGGACAGACGCCC	359bp
	12	AGCCCTCCCCAGCAGTAA	TACAGAGCGGCATCCACATC	386bp
	13	TTTTCTGGGGAGGGATCCAC	GCTGGTATGAGGCTGGGTC	284bp
	14	TGGTCCACATTCTCCACAAA	GTCCTGACGTGTTCTGAAGC	374bp
EPHB4	13	AAAAAGATGTGGATGGGAGGG	TGCTCCTGTATCCTCTCCCT	406bp
	14	TGGGCAAACAGAGTTGGAGA	ACCCAGCAGTGATGACTCTC	393bp
	14	ACAAAATTAGCTGAGGGTGG	CTGGCCCTGTATCTCAACTC	590bp

Supplemental Table 5: Oligonucleotides (5’-3’) used for site-directed mutagenesis of human *EPHB4*

c.2216G>A (p.Arg739Glu)	Forward	GAGCTACGTCCACC**<u>A</u>**AGACCTGGCTGCTC
	Reverse	GAGCAGCCAGGTCT**<u>T</u>**GGTGGACGTAGCTC
c.2345T>G (p.Ile782Ser)	Forward	AGCTCCCTGGGAGGAAAGA**<u>G</u>**TCCCATCCGA
	Reverse	TCGGATGGGA**<u>C</u>**TCTTTCCTCCCAGGGAGCT

Substituted nucleotides are highlighted in bold and underlined. All constructs were verified by Sanger sequencing.

Supplemental Videos

Supplemental Video 1: 3D reconstruction of a lymphovenous valve in E13.5 control embryo

A movie showing 3D reconstruction of confocal images of E13.5 lymphovenous valve in *Ephb4*^{wt}*;R26-mTmG*$^{+}$*;Prox1-CreER*$^{T2+}$ stained with antibodies against GFP (green), Lyve1 (red) and Prox1 (blue). Blue channel also shows weak Tomato signal from the *R26-mTmG* transgene (in all non-recombined cells). 4-OHT treatment was carried out as described in Figure 3E. Note the presence of elongated LVV leaflets extending to the lumen of the vein.

Supplemental Video 2: 3D reconstruction of a lymphovenous valve in E13.5 *Ephb4* deficient embryo

A movie showing 3D reconstruction of confocal images of E13.5 lymphovenous valve in *Ephb4*$^{flox/flox}$*;R26-mTmG*$^{+}$*;Prox1-CreER*$^{T2+}$ stained with antibodies against GFP (green), Lyve1 (red) and Prox1 (blue). Blue channel also shows weak Tomato signal from the *R26-mTmG* transgene (in all non-recombined cells). 4-OHT treatment was conducted as described in Figure 3E. Note that the LVV leaflets are collapsed.
